# Topical Application of Vancomycin Powder to Prevent Infections after Massive Bone Resection and the implantation of Megaprostheses in Orthopaedic Oncology Surgery

**DOI:** 10.5704/MOJ.2403.016

**Published:** 2024-03

**Authors:** L Andreani, E Ipponi, G Varchetta, AD Ruinato, S De-Franco, FR Campo, A D'Arienzo

**Affiliations:** Department of Orthopedics and Trauma Surgery, University of Pisa, Pisa, Italy

**Keywords:** megaprosthesis, infection, PJI, topical, vancomycin, powder

## Abstract

**Introduction:**

Periprosthetic joint infection (PJI) represents a serious burden in orthopaedic oncology. Through the years, several local expedients have been proposed to minimise the risk of periprosthetic infection. In this study, we report our outcomes using topical vancomycin powder (VP) with the aim to prevent PJIs.

**Materials and methods:**

Fifty oncological cases treated with massive bone resection and the implant of a megaprosthesis were included in our study. Among them, 22 [(GGroup A) received one gram of vancomycin powder on the surface of the implant and another gram on the surface of the muscular fascia]. The remaining 28 did not receive such a treatment (Group B). The rest of surgical procedures and the follow-up were the same for the two groups. Patients underwent periodical outpatient visits, radiographs and blood exams’ evaluations. Diagnosis of PJIs and adverse reactions to topical vancomycin were recorded.

**Results:**

None of the cases treated with topical vancomycin developed infections, whereas 6 of the 28 cases (21.4%) who did not receive the powder suffered from PJIs. These outcomes suggest that cases treated with VP had a significantly lower risk of post-operative PJI (p=0.028). None of our cases developed acute kidney failures or any other complication directly or indirectly attributable to the local administration of VP.

**Conclusions:**

The topical use of vancomycin powder on megaprosthetic surfaces and the overlying fascias, alongside with a correct endovenous antibiotic prophylaxis, can represent a promising approach in order to minimise the risk of periprosthetic infections in orthopaedic oncology surgery.

## Introduction

**Fig 1:**
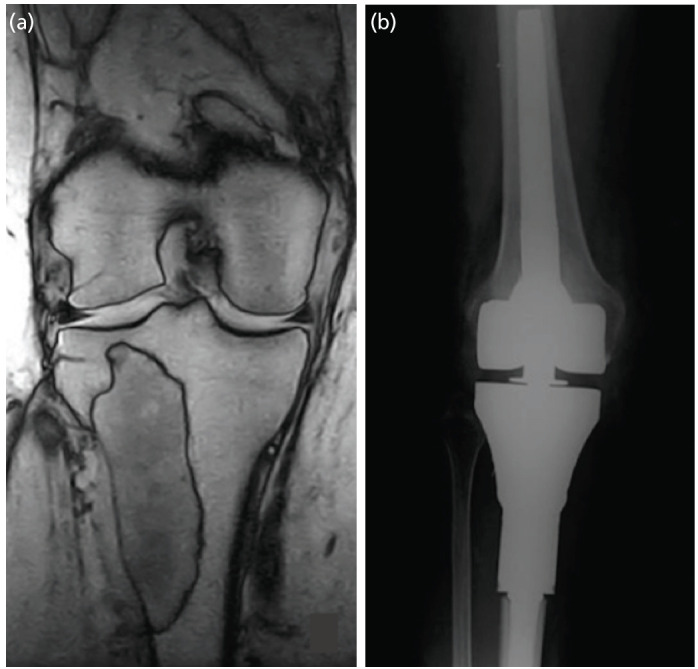
(a) Pre-operative MRI of a fibroblastic osteosarcoma treated with wide massive bone resection and (b) implant of a megaprosthetis.

Periprosthetic joint infection (PJI) represents a serious burden in orthopaedic surgery, as it occurs in 1-2% of all primary arthro-prosthetic implants^1^. This complication is even more frequent for modular megaprostheses, with a risk that can be up to more than 50% of the treated cases^2^. Megaprosthetic implants are exposed to a high risk of infections due to their large metallic surfaces that could provide an optimal substrate for bacterial colonisation^3,4^. The risk is also increased by the mean length of surgical procedures^5^. Massive bone resections, especially in case of soft tissue involvement for oncological cases, and the following reconstructive phase with the construction and the implant of the prosthetic body require longer surgical times compared to common primary arthroplasty. Furthermore, cases who suffer from malignant bone tumours, especially those who received chemotherapy, often have deficiencies in their immune systems and are therefore more exposed to the risk of post-operative infections^6,7^. All these factors, together, explain the high complication rates of infective nature that burden megaprosthetic implants in orthopaedic oncology (Fig. 1)^2^.

Through the decades, the introduction of new antibiotics and the innovation in bioengineering led the way to improvements not only in endovenous antibiotic prophylaxis^8^, but also in terms of topical antibiotic effects^3^. In recent years, several expedients have been proposed and used in order to minimise the risk of periprosthetic infections in megaprostheses. To this date, silver coated implants and antibiotic loaded cements represent the most frequently used and described in literature with some encouraging results^2,4,9-13^. The topic use of vancomycin powder, which has been reported in some papers for the prevention of PJI in arthroplasty, has not been widely experimented in bone reconstruction surgery this far^14,15^.

In this article, we report our experience with the use of vancomycin on the surfaces of both megaprosthetic implant and muscular fascia in order to minimise the risk of periprosthetic infections in orthopaedic oncology.

## Materials and Methods

This single-centre retrospective study was approved by our local ethics committee and performed in accordance with the ethical standards laid down in the 1964 Declaration of Helsinki and its later amendments.

Our study consisted of a review of all the cases that have been treated in our institution with massive resection due to a bone tumour and megaprosthetic reconstruction between June 2016 and January 2022.

For each patient, we collected their general data and data regarding their comorbidities, their histological diagnosis and the localisation of their prosthetic implants. Blood tests including C-reactive protein (CRP), white blood cell count, creatinine and albumin were also recorded and evaluated both the day before surgery and seven days after surgery for each case.

Inclusion criteria were a diagnosis of malignant (whether primary or secondary) or locally aggressive bone tumour, the implant of a megaprosthesis and the absence of a known infective process in the surgical site or in distant body segments at the moment of surgery. Exclusion criteria were an already diagnosed infection at the moment of patients’ hospitalisation, revision surgery, a pre-operative story of kidney failure or a known allergy to glycopeptides. Those cases who, after the bone resection and the implant of the prosthetic implant, were diagnosed with a local recurrence and were treated with an amputation of the affected limb have also been excluded from the study.

The megaprosthetic implant of choice was the Megasystem C [Waldemar LINK® GmbH and Co. KG, Hamburg, Germany]. Two suction subfascial drains (Redivac) were used for lower limb surgeries, while a single drain was used for upper limb interventions. All cases received the same systemic antibiotic prophylaxis. Vancomycin 1g and Tobramycin 100mg were administered intravenously every 12 hours from the night before surgery until the complete removal of surgical drains. Similar antibiotic treatments have already been used and described in previous literature^16,17^.

**Fig 2:**
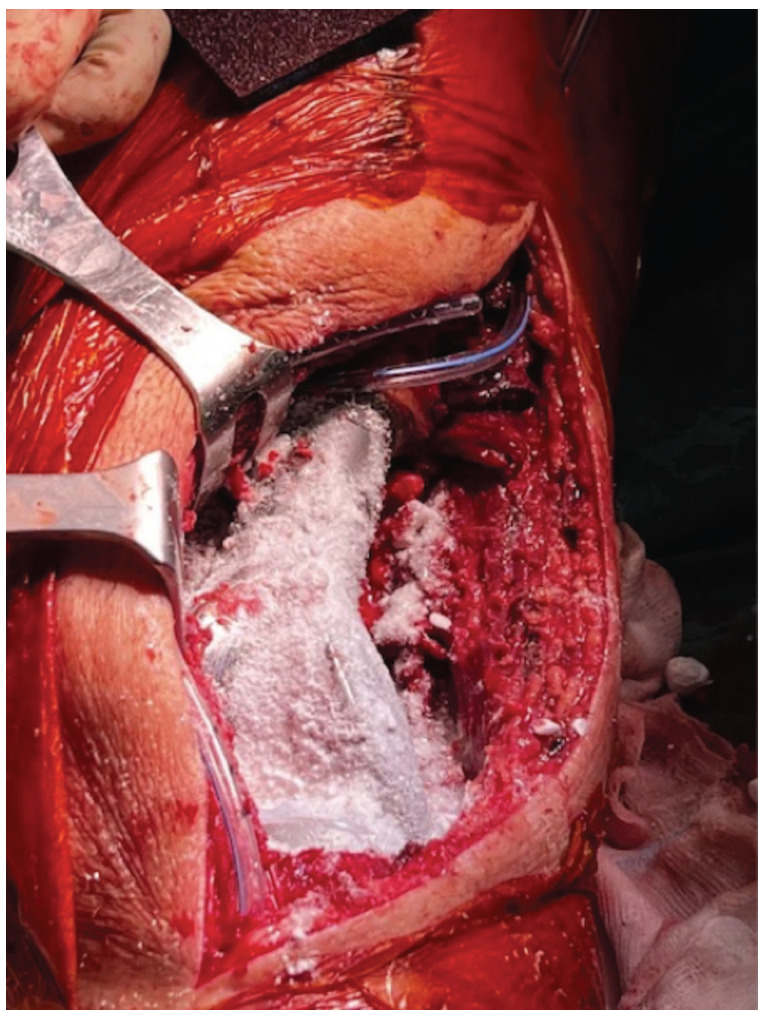
Vancomycin powder used to cover the surface of a megaprosthetic implant of knee and proximal femur.

Cases were divided into two groups depending on whether they also received or not a topic treatment with vancomycin powder. Group A received one gram of vancomycin powder directly on the surfaces of the implants and another gram on the fascial layer once it had been sutured at the end of the surgical procedure (Fig. 2). Group B did not receive any topical antibiotic treatment with vancomycin powder. Before their interventions, patients who met our pre-operative inclusion criteria did not meet the pre-operative exclusion criteria were sorted into the two groups alternating (one to one ratio; intervals of one by one) cases treated with vancomycin powder with those who did not receive the drug locally.

Post-operative follow-up consisted of serial office visits, clinical evaluations and radiographs images in order to assess their general conditions alongside with clinical and radiological outcomes of surgical treatment. When available, the clinical statements written by oncologist and general practices were evaluand for each patient, alongside with their latest blood exams. FDG-PET/TC were also performed for those who had malignant bone tumours in the context of their re-stadiation months and years after surgery. The diagnostic approaches and the final diagnoses of periprosthetic joint infection were performed in accordance with the guidelines of the American Academy of Orthopaedic Surgeons (AAOS)^18^. Each case of periprosthetic infection was recorded, along with every adverse reaction to vancomycin or post-operative complications (Grade III or higher according to the Clavien - Dindo Classification) that could be directly attributable to the drug. Statistical analysis was performed using Stata SE 13 [StataCorp LLC]. Statistical significance was set at 0.05 for all endpoints.

## Results

At their latest follow-up, fifty patients with a bone tumour who received massive bone resection and the implant of a megaprosthetic implant met our inclusion criteria and were therefore included in our study. Among them, 22 were treated with topical applications of vancomycin powder (Group A), whereas the remaining 28 cases did not have such a treatment (Group B).

Our patients’ mean age of our cases was 56.3 (14-87), 47.6 (14-87) for cases belonging to Group A and 59.1 (15-82) for those who belong to Group B. Among our 50 cases, five suffered from benign locally aggressive bone diseases, two cases had bone localisations of multiple myeloma, 25 had bone sarcomas or soft tissue sarcomas with local bone aggression and the remaining 18 cases suffered from metastatic diseases. The distribution of histological diagnoses among the two groups is portrayed in Table I.

**Table I:** Schematic resume of patients’ histological diagnosis.

Disease	Group A	Group B
Benign and locally aggressive diseases	1	4
Aneurysmal bone cyst	1	1
Giant cell tumour of bone	0	2
Benign fibrous histiocytoma of bone	0	1
Multiple myeloma	0	2
Primary malignant bone and soft tissue sarcomas	11	14
Chondrosarcoma	6	3
Ewing Sarcoma	2	3
Osteosarcoma	3	6
Synovial sarcoma	0	1
Fibromyxoid sarcoma	0	1
Metastatic Diseases	10	8
Breast carcinoma	2	1
Urothelial carcinoma	1	1
Kidney carcinoma	0	1
Stomach carcinoma	1	0
Large intestine carcinoma	1	0
Lung carcinoma	2	1
Prostatic carcinoma	0	1
Thyroid carcinoma	2	1
Melanoma	1	1
Skin carcinoma	0	1
Hematologic tumours	0	2
Multiple myeloma	0	2
Total	22	28

In our general population, 18 cases underwent a reconstruction of their hip and proximal femur. Twenty-one cases were treated around the knee joint, with prosthetic reconstruction of the distal femur (19 cases) or proximal tibia (two cases). Total femur implants were used in four cases. The remaining seven patients received a replacement of their proximal humerus. The distribution of implants’ types and locations among the two groups is summarised in Table II. Two cases (one in Group A and one in Group B) had a diagnosis of Type II Diabetes at their surgical interventions. Only one case (belonging to Group B) had made a wide use of corticosteroids before surgery, whereas none of our patients underwent chronic use of corticosteroids after surgery. Seven of our cases were smokers (3 in group A and 4 in Group B). All our cases received chemotherapy according to the EURACAN guidelines, basedon their histological diagnosis (reported in Table I)^19,20^.

**Table II:** Schematic resume of implanted prostheses, sorted per group. The number of those who suffered from an infection was mentioned within round brackets ().

Implant Site	Group A	Group B
Lower Limb	19	24
Total femur	1	3 (1)
Proximal femur and hip	9	9 (1)
Distal femur and knee	8	11 (4)
Proximal tibia and knee	1	1
Upper Limb	3	4
Proximal humerus	3	4
Total	22	28

**Fig 3:**
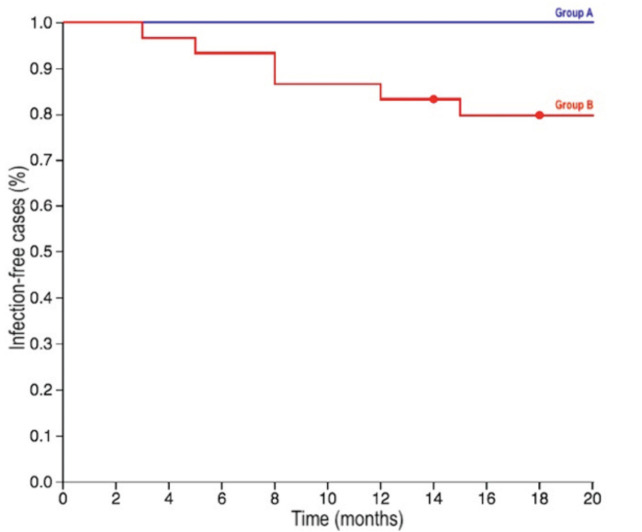
The Kaplan Meier survival curves of the two groups: Group A that received topical vancomycin powder (in blue) and Group B that did not receive the drug (in red).

The mean follow-up for our general population was 38.2 months (14-78): 32.1 (18-78) for Group A and 43.4 (14-71) for Group B. At their latest follow-up, six of our 50 patients (12.0%) had been diagnosed with a periprosthetic infection. All the six cases of infection belonged to Group B, which therefore had an infective rate of 21.4%, whereas none of the cases of Group A were diagnosed with an infection (0%). This difference was proven to be statistically significant according to an Exact Fisher Test (p=0.028), testifying the fact that, among our population, cases treated with topical vancomycin had a significantly lower risk of infection. Infections were diagnosed within three, five, eight (in two cases), 12 and 15 months after surgery (Fig. 3). The bacteria responsible for the periprosthetic infections were Meticillin-Sensitive Staphylococcus aureus (MSSAs) in three cases, Meticillin-Resistant Staphylococcus aureus (MRSAs) in two cases and Coagulase-negative Staphylococcus in another case.

The mean pre-operative and post-operative CRP, white blood cell count and creatinine values, portrayed in detail in Table III, did not significantly differ between Group A and Group B according to two tailed t-student tests. None of our patients had adverse reactions to vancomycin nor suffered from acute or chronic kidney failure through their post-operative intercourse. There was no correlation between pre-operative albumin levels and the occurrence of post-operative infections according to one-tailed t-student tests. The diabetic case included in Group B had a PJI, but the absence of other cases with the same disease did not allow further statistical evidence in this regard. Although two of the six cases with PJI were smokers, Exact fisher test did not highlight a significant correlation between the regular use of tobacco and the onset of PJIs in our whole population nor in Group B specifically.

**Table III:** Schematic resume of patients’ blood values the day before surgery (pre-op) and within seven days after surgery (post-op). The benchmarks of our laboratory were reported in square brackets []. The mean values of Group A and Group B were compared using two tailed t-student rests.

Values	Group A	Group B	Statistical Significance
CRP (pre-op) [<0.5]	1.9	2.0	p = 0.94
CRP (post-op) [<0.5]	3.4	3.6	p = 0.88
White blood cells (pre-op) [4.0 - 11.0]	6.61	6.92	p = 0.79
White blood cells (post-op) [4.0 - 11.0]	7.17	7.34	p = 0.81
Creatinine (pre-op) [0.7 - 1.2]*	0.92	0.88	p = 0.90
Creatinine (post-op) [0.7 - 1.2]*	0.93	0.90	p = 0.91
Albumin (pre-op) [3.5 - 5.5]	2.9	2.9	p = 0.98
Albumin (post-op) [3.5 - 5.5]	2.4	2.5	p = 0.95

## Discussion

Vancomycin is a glycopeptide antibiotic widely used in the treatment and prevention of gram-positive infections. Although the vast majority of reports in literature verge on its endovenous administration, a number of studies has already reported the topical use of vancomycin powder to prevent infections of surgical sites. Animal and human experiments testified the effectiveness of vancomycin powder in eradicating Staphylococcus aureus and several other common gram-positive bacteria responsible for PJIs^14,15,21-23^. Days after the administration of two grams of powder in the surgical bed, it has been shown that the topic concentration of vancomycin could be nearly 1000-fold higher than the minimum inhibitory concentration for MRSA and coagulase-negative Staphylococcus^14,15,23^. Therefore, adequate doses of the promise to be effective in preventing bacterial infections and in parallel avoid the selection of multiresistant gram-positive bacteria^14,15,23^.

These premises encouraged some institutions to use topical vancomycin powder in order to minimise the risk of infection in arthroplasty, leading to the flourishing of articles on the topic. Although the effectiveness of vancomycin in preventing surgical site infections is not unanimous to this date^24^, several authors testified a significative reduction of infection rates in cases who received a local addition of vancomycin during Total Knee Arthroplasty (TKA) or Total Hip Arthroplasty (THA)^24-31^.

So far, much less has been written about the potential role for topical vancomycin in megaprosthetic surgery, which for its nature is even more prone to periprosthetic infections. In a recent paper, Hashimoto *et al*^4^ reported their experience of five oncologic patients treated with massive bone resection and the implant of megaprostheses wrapped in vancomycin-containing cement. Despite their limited numbers, none of their cases developed any post-operative PJIs, thereby suggesting the potentiality of vancomycin as a topic prophylactic agent against bacterial infections. Although our study differs from the one by Hashimoto *et al*^4^ in terms of study design, population size and drug positioning (as they used cement while we placed vancomycin powder alone directly on the implant surface), their outcomes are in line with the ones that emerge from our cohort. In our cohort, the topical use of vancomycin powder on the surface of megaprostheses seemed to play a role in preventing PJIs attributable to gram-positive bacteria.

None of the 22 cases who received vancomycin powder on their implant surface and the overlying fascia experienced a periprosthetic infection. Comparing this result with the one of control cases, the local administration of vancomycin resulted to be associated with a significantly lower risk of periprosthetic infection, thereby testifying the effectiveness of the treatment. This outcome suggests that adding vancomycin powder during surgery, while the implant is exposed and a wide excision has been made, can prevent the colonisation and the on-site survival of gram-positive bacteria, which were responsible for all the infections diagnosed in our control group. Furthermore, in our population, the effectiveness of vancomycin powder in terms of antibiotic prophylaxis was not counterbalanced by an increase of patients’ complication rate, confirming what had already been proven in literature^14,15,21-29^. None of our cases developed any complication attributable to the drug on the surgical site nor systemically. Nephrotoxicity, which is one of the main concerns with vancomycin, was not experienced by any of our patients.

The absence of side effects apparently supported our choice to use a combination of local and intravenous vancomycin. As vancomycin has a half-life of about six hours^32^, at the end of these long and complex surgeries, its blood concentration could be dramatically reduced during the final parts of the interventions. For this reason, the local supplementation of the drug could significantly increase its concentration and dissuade the gram-positive colonisation in a crucial phase such as wound closure. Furthermore, unlike intravenous administration, topical vancomycin powder would increase drugs’ local concentration regardless of local vascularisation and without relevant systemic consequences^15^.

We are conscious that our study is not free of limitations. One of them is represented by the retrospective nature of our study, which did not allow us to perform patients’ post-operative follow-up in a condition of complete and perfect standardisation for each case. Another limitation lies in the variability of our population which included cases with several histological diagnoses and various anatomical sites. This, altogether with the consequential implant of megaprostheses that had different sizes and shapes, furtherly reduced the grade of standardisation in our cohort.

Despite these limitations, our study includes a relatively large number of cases who were treated with megaprostheses and local addition of vancomycin powder, and such a treatment resulted to be effective in order to minimise the risk of infections in our population. Although we are conscious that further studies with larger cohorts and a perspective design could provide further evidence and increase the significativeness of our assertions, our outcomes suggest that the topical use of vancomycin powder on megaprosthetic surfaces and muscular fascias could be a convenient and a relatively inexpensive option to reduce the risk of periprosthetic infections in orthopaedic oncology. The introduction of this practice in orthopaedic oncology centres could reduce the infection rates in megaprostheses, thereby promoting patients’ quality of life, increasing the mean duration of each implant and - not less important - significantly reducing the costs of revision surgery due to periprosthetic infections in a long-term scenario.

## Conclusion

In conclusion, the topical use of vancomycin powder on megaprosthetic surfaces and the overlying fascia, alongside with a correct endovenous antibiotic prophylaxis, can be effective in minimising the risk of periprosthetic infections in orthopaedic oncology surgery. It should therefore be taken in consideration as a possible alternative or addition to other local prevention methods, such as the implant of silver-coated implants or antibiotic-loaded cements.
